# Influence of Neonatal Ankyloglossia on exclusive breastfeeding in the six first months of life: a cohort study

**DOI:** 10.1590/2317-1782/20242023108en

**Published:** 2024-06-24

**Authors:** Christyann Lima Campos Batista, Alex Luiz Pozzobon Pereira

**Affiliations:** 1 Banco de Leite Humano, Hospital Universitário – HU, Universidade Federal do Maranhão – UFMA - São Luís (MA), Brasil.; 2 Programa de Pós-graduação em Odontologia, Universidade Federal do Maranhão – UFMA - São Luís (MA), Brasil.

**Keywords:** Ankyloglossia, Breastfeeding, Weaning, Cohort Studies, Prevalence, Infant

## Abstract

**Purpose:**

To analyze the influence of ankyloglossia on the prevalence and duration of exclusive breastfeeding of full-term infants up to the sixth month of life.

**Methods:**

Prospective cohort study, carried out with 225 mother-infant dyads who were followed up in the first six months of life in a center specialized in breastfeeding in a tertiary hospital. Full-term infants with asymptomatic ankyloglossia (no need for surgery) were compared with infants without change at monthly follow-up. Ankyloglossia was diagnosed using the Bristol Tongue Assessment Tool, with a positive diagnosis being considered for those with a score less than or equal to 5 considering functional and anatomical aspects. Statistical analyzes were performed using descriptive statistics, logistic regression (weaning determinants), relative risk, and survival curves (to analyze breastfeeding duration between groups with and without ankyloglossia).

**Results:**

Ankyloglossia was associated with weaning (considered even partial) before the sixth month of life. After adjusted analysis, a higher risk of weaning was detected in infants with this alteration, with a risk present from the second month of life. In the survival analysis, the duration of breastfeeding in infants with ankyloglossia was shorter when compared to children without alterations.

**Conclusion:**

Compared to infants with normal lingual frenulum, babies with ankyloglossia had shorter exclusive breastfeeding time, but well above the average observed in the general population. The risk of weaning for this group was also higher.

## INTRODUCTION

Ankyloglossia is a congenital alteration characterized by the permanence of a fibrous tissue band, caused by the failure to loosen the tongue from the buccal floor, in which the apex of the tongue is attached to a marginal resection of the alveolar ridge^(1)^. Commonly called tongue-tie, ankyloglossia occurs near the 4th week of gestational age and has an estimated prevalence of up to 8% in children younger than 1 year^(2)^.

The debate regarding the negative influence of ankyloglossia on the oral functions of newborns has gained prominence in the recent scientific literature, due to divergent opinions on this alteration’s direct relationship with breastfeeding (BF) complications, with no consensus among professionals on either the diagnosis and treatment options, or the possible anatomical and functional impairments that may arise in further stages of life^(3-5)^.

Such alteration may also have proximal impacts on breastfeeding, such as maternal pain and problems with milk supply or production for the mothers; whereas for the baby, it may cause difficulty with sucking and latching on the breast, low weight gain or early weaning^(6-10)^. These negative experiences endured by the mothers can be highly consequential for the discontinuity of breastfeeding^(11)^.

The World Health Organization (WHO) recommends to exclusively breastfeed infants until their 6th month of life, in virtue of the human milk’ critical role in the child's development, being associated with multiple health aspects, such as for the prevention of dyadic diseases, for oral health conditions like a better occlusal development, as well as acting as a preventive measure for social issues^(12)^. In low- and middle-income countries, only 37% of children under 6 months are exclusively breastfed^(13)^.

Managing children with ankyloglossia demands the correct multiprofessional monitoring in order to provide the due support to overcome the difficulties related to latching and sucking, which are frequent at the onset of lactation^(10)^. The variability in the treatment of symptomatic ankyloglossia-related outcomes expresses the complexity of tongue kinematics and infant feeding^(1,14)^.

The biomechanics of sucking during breastfeeding are a fundamental part of the child's oral skills as well as crucial for both facial and skull development, and may also compromise other aspects of life such as the individual’s breathing pattern, impair school performance and trigger spoken language disorders^(15,16)^. Therefore, it is paramount to identify the early factors that may interfere with the natural nursing process. In Brazil, early identification is mandatory since 2014, due to the enactment of a federal law^(17)^, and this assessment is part of newborn screening programs.

The association between ankyloglossia and breastfeeding dynamics remains inconclusive in the literature. Studies assess very early outcomes and do not consider the alteration's effects over time, such as the influence on exclusive breastfeeding rates that can pose risks to child development^(7,18)^. Thus, the purpose of the present research was to investigate the association between ankyloglossia and the continuity of Exclusive Breastfeeding (EBF) through a prospective follow-up in the first six months of the infant's life.

## METHOD

A prospective cohort study was conducted at the Maternal and Child Unit of the Federal University of Maranhão's University Hospital (HUUFMA - *Hospital Universitário da Universidade Federal do Maranhão, Unidade Materno-Infantil*) in the sectors of Joint Housing and Pediatric Follow-up Outpatient Clinic at the Human Milk Bank (BLH - *Banco de Leite Humano*), a center specialized in breastfeeding difficulties. HUUFMA is located in the city of São Luís, a state capital in northeastern Brazil. It is a highly complex referral hospital for high-risk and low-risk pregnancies. The estimated population of São Luís is 1,101,884, with an average income of 3.2 minimum wages. The data collection period ranged from January 2019 to December 2021.

To calculate the sample size, was considered the alteration level of the lingual frenulum as reported in a previous prevalence study (8%)^(2)^. Reckoning that in 2018, 15,959 live births were tallied in the city where the study was devised, the minimum number of computed participants was 113 subjects for a 95% confidence level (population study with 80% power, 5% error). A total of 329 mother-infant dyads met the baseline inclusion criteria. However, only 225 participants remained in the study's follow-up, in accordance with the inclusion and exclusion criteria.

Mothers who expressed a desire to breastfeed, without any medical contraindications for natural nursing, were included in the study. Infants with heart diseases, lung conditions, neuropathies or congenital genetic syndromes were not included, as well as twins, preterm newborns and infants weighing less than 2kg at birth.

Participants were recruited while the speech-language assessment was being conducted in the hospital's Joint Housing, after 48 hours of birth, over the course of the newborn screenings. The inclusion of individuals with ankyloglossia was accomplished during the Neonatal Tongue Screening Test (*Teste da Linguinha*). Ankyloglossia was diagnosed by specialized and qualified speech-language pathologist (4 professionals with more than 5 years of experience in breastfeeding participated in the assessments during the study period), specifically trained to conduct such evaluations. Was applied the Bristol Tongue Assessment Tool – BTAT^(19)^, which is based on the assessment of 4 aspects, 2 anatomical (tongue apex appearance and fixation in the alveolus) and 2 functional (tongue elevation and protrusion), being considered altered in cases scoring less than or equal to 3 out of a maximum score of 8. The comparison group (infants without ankyloglossia, with a BTAT score equal to or greater than 6) were composed of participants attending routine follow-up at the Human Milk Bank during the period in which newborns with ankyloglossia were being monitored.

Regarding the individuals with ankyloglossia, were only included in the study EBF infants presenting no indication of breastfeeding difficulties, that is, with asymptomatic alterations. Babies who for some reason fitted the indication criteria for surgical correction of ankyloglossia were excluded from the study and referred for the procedure, to avoid the worst outcome of non-intervention, which would be weaning itself. The surgical indicators were: nursing mothers with breastfeeding pain, presence of nipple trauma, low milk production, low milk intake by the infant (hypoglycemia). All participants were assisted by the BLH’s multidisciplinary team to overcome breastfeeding difficulties and were properly instructed on the management of the nutritional aspect by a pediatrician along with a nutritionist.

The dependent variable that assessed weaning (babies who were no longer in EBF) was collected during the monthly appointments through an interview with the mothers. The infant's mother was then inquired how the baby was feeding at that moment. At the end of the follow-up, a second numerical outcome variable was produced, regarding how many months the infant remained exclusively in the mother's breast. When the infants were no longer on EBF it was considered weaning, even if they were on mixed or partial breastfeeding.

Social and demographic data were collected at the first month appointment, when the participants visited the BLH for their follow-up in the cohort. Birth-related variables were collected in the live birth certificate. After the first appointment, the infants and their mothers had a monthly check-up with a pediatrician and a speech-language pathologist, where they were routinely guided and assisted to maintain breastfeeding.

The present study was approved by the Research Ethics Committee of the HUUFMA and registered under No. 3,052,208. All of the newborns' mothers and/or guardians were informed about the purpose, risks, benefits and procedures of this research and signed the Informed Consent Form to participate in this study.

Descriptive statistics was performed through mean and standard deviation for variables with normal distribution, median and interquartile range for the other numerical variables and through frequency and percentage for categorical variables. The verification of the numerical variables’ normal distribution was achieved using the Shapiro-Wilk test. The association analysis between the dependent and independent variables was carried out using the Student's t-test or the Mann-Whitney U test when indicated in the tables. For the categorical variables, the Chi-square or Fisher's exact test was applied in conformance with the tables.

The odds ratio for the early weaning event was calculated inputting only the variables that presented p < 0.20 in the association tables for the univariate model. Some categorical data were analyzed as dummy variables, and just the one with the highest chance was considered. The adjusted model selection was established through the Forward Stepwise Regression method, defining the variable as an input criterion in the model with α^1^ = 0.100 and output with α^2^ = 0.050. The relative risk was calculated in the EBF prevalence analysis according to the month. To evaluate the weaning time for the groups with and without ankyloglossia, the Kaplan-Meier survival curve was applied. The adopted significance level for all tests was 5% (p<0.05). The analyses were conducted in the IBM SPSS Statistics program (version 26).

## RESULTS

The total of 225 dyads contained the information of the outcome variable (weaning) in the study database and entered the final analysis. The maternal age mean was 28 years (22-34 IQR). It was observed that 27% of the sample presented early weaning and that, among infants with ankyloglossia, which represented 24% of the sample, the proportion of weaning increased to 51.9%. Ankyloglossia was associated with weaning (p < 0.001).

Furthermore, Table 1 indicates that both the newborn sex (p = 0.040) and the maternal occupation (p = 0.039) were also associated with weaning, with a higher proportion of weaning among male babies (68.8%) and working mothers (58.3%). The assessed birth measures were considered adequate for full-term babies and none of them were associated with weaning before the infant's sixth month of life (p>0.05).

**Table 1:** Correlation of the social and demographic characteristics of mothers and infants along with its association with weaning before the 6th month of infants, 2019-2021

Variables	No (%)	Weaning before 6 months		*P*-value
		No	Yes	
		No (%)	No (%)	
Newborn Sex				**0.040**
Male	130 (57.8)	88 (53.7)	42 (68.8)	
Female	95 (42.2)	76 (46.3)	19 (31.2)	
Breastfed in the 1st hour				0.329
Yes	138 (65.4)	104 (67.5)	34 (59.6)	
No	73 (34.6)	50 (32.5)	23 (40.4)	
Missing (14)				
Maternal Age				0.311
Under 18 years	18 (8.1)	12 (7.5)	6 (9.8)	
18 to 30 years	117 (52.7)	81 (50.3)	36 (59)	
Over 31 years	87 (39.2)	68 (42.2)	19 (31.1)	
Missing (3)				
Marital Status				0.189
With partner	179 (79.6)	134 (81.7)	45 (73.8)	
Without partner	46 (20.4)	30 (18.3)	16 (26.2)	
Maternal Education				0.912
Up to Elementary School	31 (13.8)	22 (13.4)	9 (14.8)	
High School	142 (63.1)	103 (62.8)	39 (63.9)	
Higher Education or more	52 (23.1)	39 (23.8)	13 (21.3)	
Maternal Occupation				**0.039**
Unemployed	104 (47.1)	81 (50.3)	23 (38.3)	
Working	100 (45.2)	65 (40.4)	35 (58.3)	
Studying	17 (7.7)	15 (9.3)	2 (7.7)	
Missing (4)				
Family Income				0.101^1^
1 minimum wage (MW) or less	94 (42.2)	75 (46.3)	19 (31.1)	
Above 1 to 3 MW	80 (35.9)	57 (35.2)	23 (37.7)	
Above 3 to 5 MW	43 (19.3)	26 (16)	17 (27.9)	
Above 5 MW	6 (2.7)	4 (2.5)	2 (3.3)	
Missing (2)				
Primiparity				0.076
Yes	111 (49.3)	75 (45.7)	36 (59)	
No	114 (50.7)	89 (54.3)	25 (41)	
Type of Delivery				0.901
Vaginal	103 (46)	75 (45.7)	28 (46.7)	
Cesarean	121 (54)	89 (54.3)	32 (53.3)	
Missing (1)				
Ankyloglossia				**<0.001**
Yes	54 (24)	26 (15.9)	28 (45.9)	
No	171 (76)	138 (84.1)	33 (54.1)	
NB data at birth	Median (IQR) or Mean (SD)			
Apgar Score				
1-minute^*^	9 (8-9)	9 (8-9)	9 (8-9)	0.359^2^
5-minute*	9 (9-9)	9 (9-9)	9 (9-9)	0.052^2^
Birth weight^**^	3212.9 (27)	3243.8 (39.1)	3197.4 (64)	0.537^3^
Cephalic perimeter*	34.5 (33.5-35.3)	34.5 (33.5-35.5)	34.1 (33.5-35)	0.981^2^
Chest girth**	32.8 (0.3)	32.9 (0.4)	33.1 (0.4)	0.869^3^
Length*	49 (47-50)	49 (47-50)	48.5 (47-49.5)	0.385^2^

* Presented in median, 25-75 percentile range;

** mean and standard deviation

1 Fisher's Exact Test, further categorical variables analyzed by Pearson's Chi-Square Test;

2 Mann-Whitney U Test;

3 Student's T-Test

The newborn's sex, as well as the maternal occupation, family income and ankyloglossia were associated with weaning. However, after adjusting the variables, it was observed that only the presence of ankyloglossia remained associated with weaning. The chance of weaning in this group was almost 4.5 times higher when compared to infants without the alteration (p < 0.001 OR = 4.49 95% CI = [2.29;8.80]). Being female presented a p-value of exactly 0.050, as shown in Table 2, and was not considered as a protective factor due to exhibiting a confidence interval variation very close to 1.

**Table 2:** Unadjusted and adjusted logistic regression analysis using exclusive breastfeeding in the 6th month as the outcome variable and the expository variables, 2019-2021

	Univariate Analysis		Adjusted Analysis	
	P-Value	OR (95% CI)	P-Value	OR (95% CI)
Sex, female	0.042	0.52 (0.28-0.97)	0.050	0.50 (0.25-0.99)
Marital status, without partner	0.192	1.58 (0.79-3.18)		
Occupation, working	0.043	1.89 (1.02-3.52)		
Income, between 3 and 5 MW	0.019	2.58 (1.16-5.69)		
Primiparity	0.078	0.58 (0.32-1.06)		
5-minute Apgar	0.174	0.62 (0.31-1.23)		
Presence of Ankyloglossia	<0.001	4.50 (2.33-8.67)	<0.001	4.49 (2.29-8.80)

Adjusted model selection through the Forward Stepwise Regression method, model input with α^1^=0.100 and output α^2^=0.050. Variables removed from the model: marital status (p = 0.154), occupation (0.309), Apgar 5 (p = 0.338), family income (0.930) and primiparity (p = 0.531). Model adjustment indicators p < 0.001, R^2^ 0.0981

**Caption:** OR (odds ratio), CI (confidence interval). MW (minimum wages)

Table 3 shows the relative risk for weaning per month. It was ascertained that from the second month onwards, children with ankyloglossia had a higher risk of not being in EBF. Predominantly, the proportion of EBF in infants with the alteration was always lower at all months when compared to infants without the alteration.

**Table 3:** Prevalence and Risk estimates of the study participants’ Exclusive Breastfeeding, categorized according to ankyloglossia, 2019-2021

	No (%)	Ankyloglossia		RR (95% CI)
		No	Yes	P-value
		No (%)	No (%)	
**Reported EBF**				
1st month	199 (93)	161 (94.7)	38 (86.4)	2.5 (0.9-6.8)
				0.058
2nd month	157 (89.7)	134 (93.1)	23 (74.2)	3.7 (1.5-8.6)
				0.002
3rd month	146 (88.5)	124 (94.7)	22 (64.7)	6.6 (28-15.4)
				<0.001
4th month	146 (86.9)	125 (91.9)	21 (65.6)	4.25 (2.0-8.9)
				<0.001
5th month	126 (83.4)	111 (88.8)	15 (57.7)	3.7 (1.9-7.3)
				<0.001
6th month	95 (49.7)	84 (56.8)	11 (25.6)	1.7 (1.3-2.2)
				<0.001

**Figure 1:**
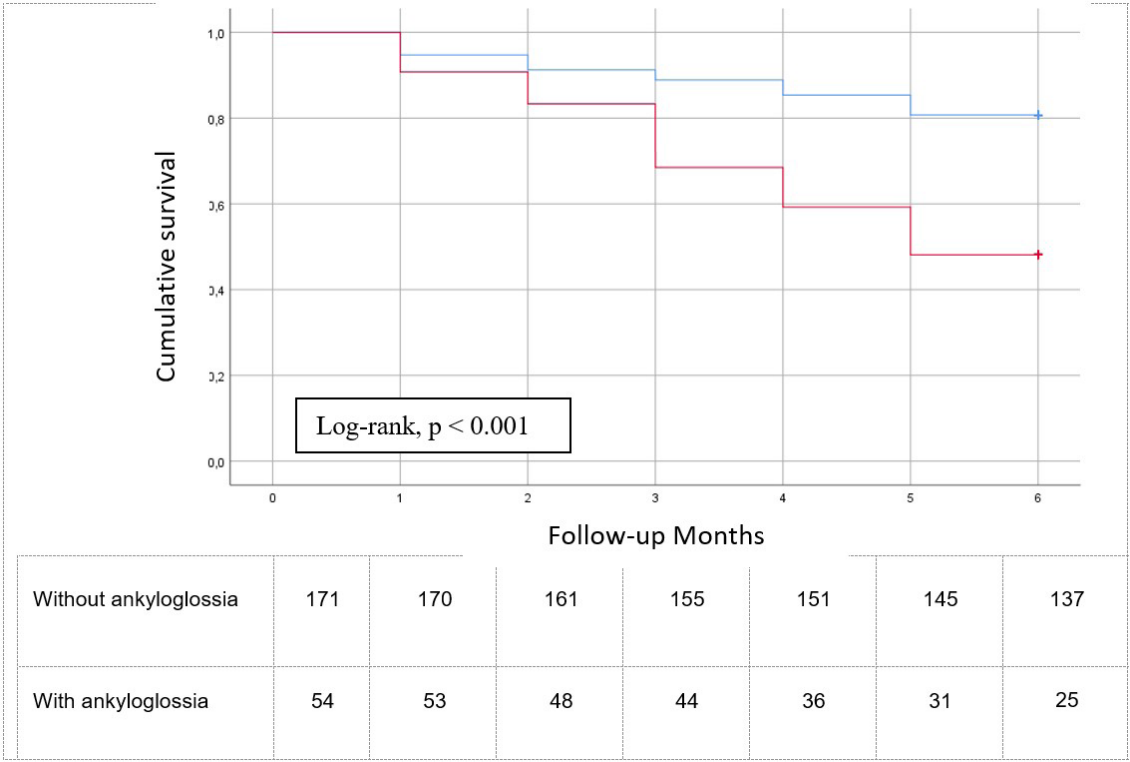
Kaplan-Meier curves representing the weaning time according to the presence or absence of ankyloglossia. Infants with ankyloglossia had a worse time of weaning than individuals who did not have ankyloglossia (p < 0.001). The exclusive breastfeeding mean (with a 95% confidence interval) for the group without ankyloglossia was 5.40 (5.2 - 5.6) months; and for the group with ankyloglossia was 4.50 (4.03 - 4.97)

**Caption:** RR – Relative Risk, CI – Confidence Interval, EBF – Exclusive breastfeeding,

Figure 1 depicts the difference between the two groups analyzed with the Kaplan-Meier curves, demonstrating that children with ankyloglossia had shorter breastfeeding time (log-rank p < 0.001) with a mean of 4.5 months (95%CI = 4.03;4.97) of the EBF period. For the group without the alteration, the mean was 5.4 months (95% CI 5.2-5.6).

## DISCUSSION

The main findings of the present study designed for the assessment of the ankyloglossia’s influence on the EBF aspects suggest that this alteration is associated with weaning closer to birth in diagnosed healthy full-term infants, when compared to children without the alteration.

The influence of ankyloglossia on the breastfeeding time as reported herein is consistent with other similar data in the medical literature. A study conducted in Spain with 1102 newborns detected a higher prevalence of alterations in male infants and the exclusive breastfeeding rate was 50.35%^(20)^. A prospective study carried out in London reported a 49% rate of EBF in infants with ankyloglossia at a 3-month follow-up^(21)^. Higher rates of adherence to exclusive breastfeeding, such as 66.6% at 3 months, were registered in cases where infants were previously submitted to corrective surgical procedures^(22)^. Observational approaches indicate that the EBF rates are similar to those found in the present research.

Another study reported that EBF rates can be similar even without considering interventions to correct the alteration^(23)^. It should be noted that in the first weeks of the infant's life, the presence of ankyloglossia is concomitant with other lactation-related changes^(18)^. Studies further state that the presence of the alteration may be related to a negative perception of the mother when breastfeeding, which may lead to uncertainty regarding which factor may be generating the difficulties experienced in the first weeks of natural nursing^(24)^.

Several factors seem to mediate the relationship between the lingual frenulum alteration and early weaning, since it is concomitant with other early issues such as pain and difficulty in handling the infant, which are associated with breastfeeding in the first weeks of life and may hinder the establishment of the newborns’ natural nursing. Nevertheless, these factors can be related to other previous conditions such as cesarean delivery and lack of previous breastfeeding experience^(7,18,25,26)^.

Regarding the present study, it is important to emphasize that the association between ankyloglossia and weaning was present as early as the second month of the child's life, a critical period in which the abandonment of exclusive breastfeeding is often referred to due to the return of mothers to work^(27)^. In Brazil, most women assisted by basic labor laws are entitled to maternity leave with remuneration until the fourth month of postpartum, although it should be taken into consideration that informal work is rather frequent.

According to the Brazilian National Survey on Child Nutrition (ENANI - *Estudo Nacional de Alimentação e Nutrição Infantil*)^(28)^, the prevalence of EBF in Brazil has shown a growth trend, reaching 60% of children under 4 months and 45.7% of children under 6 months. However, the median duration of EBF was 54.1 days (1.8 months) according to the latest breastfeeding prevalence survey^(29)^. The breastfeeding indicators reported herein demonstrate the importance of providing support to women, targeting the identification of mothers at risk of early discontinuation^(30)^. The activities practiced in the milk banks seem to have a positive effect on promoting natural nursing and aiding babies with breastfeeding difficulties^(31)^.

The present study was conducted at a specialized breastfeeding center, which may lead to a selection bias or reported rates, as mothers were continuously instructed to continue exclusive breastfeeding and were assisted when they reported problems. That being said, this circumstance can be seen as a research strength, even for the group with the alteration, since the total breastfeeding time was longer than the national average, demonstrating that adequate monitoring and guidance can be decisive for reducing the chance of weaning, therefore evidencing the clinical and non-surgical measures as a protective effect of breastfeeding.

Frenotomy has been pointed out as the standard treatment for the positive diagnosis of this alteration with symptomatic cases, mainly reducing the mother's perceived pain^(32)^. Still, it is a consensus among health professionals who deal with breastfeeding that an accurate clinical assessment needs to be conducted, based on the best scientific protocols^(19,33)^, in order to avoid unnecessary procedures and prevent iatrogenesis^(5)^.

Another limitation of the present study is related to the patients' follow-up, since the voluntary participation and the socioeconomic level of the mothers may have suppressed a greater attendance to the follow-up appointments. In addition, dyads that were successfully breastfeeding may not have felt the need to attend the follow-up, even if they had a scheduled medical appointment. Further studies should be encouraged to establish the late impact of the tongue's anatomical alteration along with the assessment of objective indicators to verify sucking and swallowing impairments in these infants. It is noteworthy that the present study could serve as a guideline for other observations that take into consideration the factors that can be associated with ankyloglossia and that may potentially interfere with breastfeeding. We also emphasize that it is imperative that the speech-language pathologist acts as an indispensable part of the multidisciplinary team, as they are the qualified professionals to evaluate and treat the oral dysfunctions present in breastfeeding.

## CONCLUSION

In the present study, neonatal ankyloglossia was shown to have an association with feeding in term infants with asymptomatic ankyloglossia (without the need for intervention) when compared to children with normal lingual frenulum, reducing the time of exclusive breastfeeding and increasing the risk of weaning.
