# Supplementary material for: Influence of Neonatal Ankyloglossia on exclusive breastfeeding in the six first months of life: a cohort study
Source: Codas. 2024 Jun 24;36(3):e20230108. doi: 10.1590/2317-1782/20242023108en (PMC467001; doi:10.1590/2317-1782/20242023108en)
Supplement: Translation

# Translation: Influência da Anquiloglossia neonatal na amamentação exclusiva nos seis primeiros meses de vida: estudo de coorte

**DOI:** 10.1590/2317-1782/20242023108pt

**Authors:** Christyann Lima Campos Batista, Alex Luiz Pozzobon Pereira

**Affiliations:** 1 Banco de Leite Humano, Hospital Universitário – HU, Universidade Federal do Maranhão – UFMA - São Luís (MA), Brasil.; 2 Programa de Pós-graduação em Odontologia, Universidade Federal do Maranhão – UFMA - São Luís (MA), Brasil.

**Keywords:** Anquiloglossia, Aleitamento Materno, Desmame, Estudos de Coortes, Prevalência, Lactente

## Abstract

**Objetivo:**

Analisar a influência da anquiloglossia na prevalência e no tempo de exclusividade do aleitamento materno de lactentes a termo até o sexto mês de vida.

**Métodos:**

Estudo de coorte prospectivo, realizado com 225 díades mãe-bebê que foram acompanhadas nos seis primeiros meses de vida em centro especializado em amamentação em um hospital terciário. Lactentes a termo com anquiloglossia do tipo assintomática (sem necessidade de cirurgia) foram comparados com lactentes sem alteração em um acompanhamento mensal. O diagnóstico de anquiloglossia foi realizado através do Bristol Tongue Assessment Tool, sendo considerados com diagnóstico positivo aqueles com escore menores ou iguais a 5 considerando os aspectos funcionais e anatômicos. As análises estatísticas foram realizadas através de estatística descritiva, regressão logística (determinantes do desmame), risco relativo e curvas de sobrevivência (para analisar o tempo de aleitamento entre os grupos com e sem anquiloglossia).

**Resultados:**

A anquiloglossia esteve associada com o desmame (considerado ainda que parcial) antes do sexto mês de vida. Após análise ajustada, foi detectado maior risco de desmame nos lactentes com a alteração presente, com risco presente a partir do segundo mês de vida. Na análise de sobrevida, o tempo de aleitamento nos lactentes com anquiloglossia foi menor quando comparadas às crianças sem alteração.

**Conclusão:**

Em comparação com lactentes com freio lingual normal, os bebês com anquiloglossia apresentaram tempo menor de aleitamento exclusivo, porém bem acima da média observada na população geral. O risco de desmame para este grupo também foi maior.

## INTRODUÇÃO

A anquiloglossia é uma alteração congênita caracterizada pela permanência de uma banda mucosa fibrosa, gerada pela falha de soltura da língua do soalho bucal, em que a ponta da língua fica presa em porção marginal à crista alveolar^(1)^. Comumente chamada de língua presa, a anquiloglossia ocorre próximo a 4ª semana de idade gestacional e possui uma prevalência estimada de até 8% em crianças menores de 1 ano^(2)^.

O debate sobre a influência negativa da anquiloglossia nas funções orais dos neonatos tem ganhado destaque na literatura científica recente, devido a visões divergentes sobre a relação direta da alteração com as dificuldades no aleitamento materno (AM), falta de consenso entre profissionais sobre o diagnóstico e opções de tratamento e possíveis desequilíbrios anatômicos e funcionais que podem surgir em outras fases da vida^(3-5)^.

A alteração também pode trazer impactos proximais no aleitamento materno, como, por exemplo, para as mães dor materna, problemas de suprimento ou produção de leite; e para o bebê dificuldade de sucção e pega no seio, baixo ganho de peso e desmame precoce^(6-10)^. Essas experiências negativas vivenciadas pelas mães podem ter papel decisivo na descontinuidade da amamentação^(11)^.

A Organização Mundial da Saúde (OMS) recomenda que o aleitamento materno deve ser exclusivo até o 6º mês de vida, devido ao decisivo papel do leite humano no desenvolvimento da criança nos mais diversos aspectos de saúde, para a prevenção de doenças para a díade, para a saúde bucal como melhor desenvolvimento oclusal, atuando também como fator preventivo de problemas sociais^(12)^. Em países de baixa e média renda, apenas 37% das crianças menores de 6 meses são exclusivamente amamentadas^(13)^.

O manejo da criança com anquiloglossia passa pelo correto acompanhamento multiprofissional para o suporte na superação das dificuldades relacionadas com a pega e sucção, que são comuns no início da lactação^(10)^. A variabilidade no tratamento dos desfechos relacionados à anquiloglossia sintomática indica a complexidade da movimentação lingual e da alimentação do lactente^(1,14)^.

Os mecanismos de sucção desempenhados na amamentação são parte fundamental das habilidades orais e têm papel decisivo para o desenvolvimento da face e do crânio, podendo também trazer prejuízos em outros aspectos da vida como o padrão respiratório, o desempenho escolar e disfunções de linguagem oral^(15,16)^. Por isso, é fundamental a identificação de fatores precoces que possam interferir na prática da amamentação natural. No Brasil, a identificação precoce se tornou obrigatória a partir de 2014 com a promulgação de uma lei federal^(17)^, sendo esse rastreamento parte dos programas de triagens neonatais.

Permanece inconclusiva na literatura a relação da anquiloglossia com a dinâmica do aleitamento materno. Os estudos avaliam desfechos muito precoces e não consideram os efeitos da alteração ao longo do tempo, como a influência nas taxas de amamentação exclusiva que podem trazer riscos para o desenvolvimento da criança^(7,18)^. Portanto, o objetivo desta pesquisa foi investigar a associação entre a anquiloglossia e a continuidade do Aleitamento Materno Exclusivo (AME) através de um seguimento prospectivo nos primeiros seis meses de vida.

## MÉTODO

Foi conduzido um estudo do tipo coorte prospectiva realizado no Hospital Universitário da Universidade Federal do Maranhão (Unidade Materno-Infantil) – HUUFMA nos setores de Alojamento Conjunto e Ambulatório de Seguimento Pediátrico no Banco de Leite Humano (BLH), centro especializado em dificuldades no aleitamento. O HUUFMA encontra-se localizado na cidade de São Luís, capital de um estado do nordeste brasileiro. É um hospital de alta complexidade, referência para gravidez de risco alto e habitual. A população estimada de São Luís é de 1.101.884 e renda média de 3,2 salários-mínimos. O período de coleta dos dados deu-se entre janeiro de 2019 a dezembro de 2021.

Para o cálculo do tamanho da amostra, foi considerado o nível de alteração do frênulo lingual relatado em estudo de prevalência anterior (8%)^(2)^. Considerando que no ano de 2018 foram computados 15959 nascidos vivos na cidade de realização do estudo, o número mínimo de participante calculado foi de 113 participantes para um nível de confiança de 95% (estudo populacional com poder de 80%, erro de 5%). O total de 329 díades mãe-bebê preencheram os critérios de inclusão no *baseline*. Entretanto, 225 permaneceram acompanhados no estudo, obedecidos os critérios de inclusão e exclusão.

Foram incluídas no estudo as mães que manifestaram vontade de amamentar, sem contraindicações médicas para amamentação. Não foram incluídos bebês cardiopatas, pneumopatas, neuropatas ou com síndromes genéticas congênitas, além de gemelares, recém-nascidos pré-termo e aqueles que tinham peso ao nascer menor que 2kg.

O recrutamento dos participantes aconteceu no momento da avaliação fonoaudiológica realizada no Alojamento Conjunto do hospital, após 48 horas de nascimento, no momento das triagens neonatais. A inclusão dos participantes com anquiloglossia foi realizada no momento do Teste da Linguinha. O diagnóstico da anquiloglossia foi realizado por fonoaudiólogos especializados e treinados (4 profissionais com mais de 5 anos de experiência em aleitamento participaram das avaliações no período do estudo), com treinamento específico para realização das avaliações. Foi utilizado o *Bristol Tongue Assessment Tool* – BTAT^(19)^ que é baseado na avaliação em 4 aspectos, sendo 2 anatômicos (aparência da ponta da língua e fixação no alvéolo) e 2 funcionais (elevação e protrusão da língua), sendo considerado alterados para escores menores ou iguais a 3 de uma pontuação máxima de 8. O grupo de comparação (lactentes sem anquiloglossia, que tinham BTAT igual ou maior que 6) foram de lactentes que estavam nas consultas de rotina no Banco de Leite Humano no período em que os lactentes com anquiloglossia eram acompanhados.

Para bebês com anquiloglossia, somente foram incluídos no estudo os lactentes que estavam em AME e que não possuíam indicativo de dificuldades na amamentação, ou seja com alteração do tipo assintomática. Os bebês que por algum motivo apresentaram critérios para indicação cirúrgica de correção da anquiloglossia foram removidos do estudo e encaminhados para realização do procedimento, para evitar o pior desfecho da não-intervenção que seria o próprio desmame. Os indicadores para cirurgia eram: nutrizes com dor ao amamentar, presença de traumas mamilares, baixa produção de leite, baixa ingesta de leite pelo lactente (hipoglicemia). Todos os participantes contaram com assistência da equipe multiprofissional do BLH para superação das dificuldades na amamentação e foram devidamente orientados sobre o manejo do aspecto nutricional por médico pediatra e nutricionista.

A variável dependente que avaliou o desmame (bebês que não estavam mais em AME) foi coletada nas consultas mensais na entrevista com as mães. Era questionado à mãe do bebê como estava a alimentação do bebê naquele momento. Ao final do seguimento, foi realizada uma segunda variável desfecho numérica que era por quantos meses o lactente permaneceu somente no seio materno. O desmame foi considerado quando os lactentes não estavam mais em AME, ainda que estivessem em aleitamento misto ou parcial.

Os dados sociais e demográficos foram coletados na consulta do primeiro mês, quando os lactentes compareceram no BLH para consulta de seguimento na coorte. As variáveis relativas ao nascimento foram coletadas na declaração de nascido vivo. A partir da primeira consulta, os lactentes e suas mães realizavam uma consulta mensal com médico pediatra e fonoaudiólogo, onde eram rotineiramente orientados e auxiliados a manter a amamentação.

Este estudo foi aprovado pelo Comitê de Ética em Pesquisa do HUUFMA sob o parecer nº 3.052.208. Todas as mães e/ou responsáveis pelos recém-nascidos foram informados sobre os objetivos, riscos, benefícios e procedimentos da pesquisa e assinaram o Termo de Consentimento Livre e Esclarecido para participarem do estudo.

A estatística descritiva foi realizada através de média e desvio padrão para variáveis com distribuição normal, mediana e intervalo interquartil para as demais variáveis numéricas e através de frequência e porcentagem para variáveis categóricas. A verificação da normalidade de distribuição das variáveis numéricas foi realizada através do teste de Shapiro-Wilk. A análise de associação entre as variáveis dependentes e independentes foi realizada através do teste *T de Student* ou do teste *U de Mann-Whitney* quando indicado nas tabelas. Para as variáveis categóricas, foi utilizado teste Qui-quadrado ou Exato de Fisher quando indicado nas tabelas.

Foi realizado o cálculo da razão de chances para o evento desmame precoce, sendo consideradas as variáveis para o modelo univariado apenas as que apresentaram p < 0,20 nas tabelas de associação. Algumas variáveis foram analisadas como categóricas *dummy*, sendo apresentada somente a com maior chance. A seleção para modelo ajustado aconteceu através do método *Stepwise forward,* definindo-se como critério de entrada da variável no modelo com α^1^ = 0,100 e de saída α^2^ = 0,050. Na análise da prevalência do AME de acordo com mês foi calculado o risco relativo. Para analisar o tempo de desmame referente aos grupos com e sem anquiloglossia, foi adotada a curva de sobrevivência de Kaplan-Meier. O nível de significância adotado para todos os testes foi de 5% (p<0,05). As análises foram conduzidas no programa IBM SPSS Statistics (versão 26).

## RESULTADOS

O total de 225 díades continha a informação da variável desfecho (desmame) no banco de dados do estudo e entraram na análise final. A mediana da idade materna foi de 28 anos (IQ 22-34). Observou-se que 27% da amostra apresentou desmame precoce e que, entre os lactentes com anquiloglossia, que representaram 24% da amostra, a proporção de desmame subiu para 51,9%. A anquiloglossia apresentou associação com o desmame (p < 0,001).

Na Tabela 1, observa-se ainda que o sexo do recém-nascido (p = 0,040) e a ocupação materna (p = 0,039) também apresentaram associação com o desmame, sendo observada uma proporção maior de desmame entre os bebês do sexo masculino (68,8%) e nas mães que trabalham (58,3%). As medidas de nascimento apresentadas foram consideradas adequadas para bebês a termo e nenhuma delas apresentou associação com o desmame antes do sexto mês de vida (p>0,05).

**Tabela 1:** Associação das características sociais e demográficas das mães e dos lactentes e sua associação com o desmame antes do 6º mês dos lactentes, 2019-2021

Variáveis	N (%)	Desmame antes do 6º mês		Valor de *P*
		Não	Sim	
		N (%)	N (%)	
Sexo do Recém-Nascido				**0,040**
Masculino	130 (57,8)	88 (53,7)	42 (68,8)	
Feminino	95 (42,2)	76 (46,3)	19 (31,2)	
Amamentou na 1ª hora				0,329
Sim	138 (65,4)	104 (67,5)	34 (59,6)	
Não	73 (34,6)	50 (32,5)	23 (40,4)	
*Missing (14)*				
Idade materna				0,311
Menor que 18 anos	18 (8,1)	12 (7,5)	6 (9,8)	
18 até 30 anos	117 (52,7)	81 (50,3)	36 (59)	
Acima de 31 anos	87 (39,2)	68 (42,2)	19 (31,1)	
*Missing (3)*				
Estado Civil				0,189
Com companheiro	179 (79,6)	134 (81,7)	45 (73,8)	
Sem companheiro	46 (20,4)	30 (18,3)	16 (26,2)	
Escolaridade Materna				0,912
Até o Ens. Fund.	31 (13,8)	22 (13,4)	9 (14,8)	
Ens. Médio	142 (63,1)	103 (62,8)	39 (63,9)	
Ens. Superior ou mais	52 (23,1)	39 (23,8)	13 (21,3)	
Ocupação materna				**0,039**
Sem trabalho	104 (47,1)	81 (50,3)	23 (38,3)	
Trabalha	100 (45,2)	65 (40,4)	35 (58,3)	
Estudante	17 (7,7)	15 (9,3)	2 (7,7)	
*Missing (4)*				
Renda Familiar				0,101^1^
1 salário-mínimo (SM) ou menos	94 (42,2)	75 (46,3)	19 (31,1)	
Acima de 1 até 3 SM	80 (35,9)	57 (35,2)	23 (37,7)	
Acima de 3 até 5 SM	43 (19,3)	26 (16)	17 (27,9)	
Acima de 5 SM	6 (2,7)	4 (2,5)	2 (3,3)	
*Missing (2)*				
Primiparidade				0,076
Sim	111 (49,3)	75 (45,7)	36 (59)	
Não	114 (50,7)	89 (54,3)	25 (41)	
Tipo de parto				0,901
Vaginal	103 (46)	75 (45,7)	28 (46,7)	
Cesáreo	121 (54)	89 (54,3)	32 (53,3)	
*Missing (1)*				
Anquiloglossia				**<0,001**
Sim	54 (24)	26 (15,9)	28 (45,9)	
Não	171 (76)	138 (84,1)	33 (54,1)	
Dados do RN ao nascer	Mediana (IQ) ou Média (DP)			
Apgar Score				
1º minuto*	9 (8-9)	9 (8-9)	9 (8-9)	0,359^2^
5º minuto*	9 (9-9)	9 (9-9)	9 (9-9)	0,052^2^
Peso ao nascer**	3212,9 (27)	3243,8 (39,1)	3197,4 (64)	0,537^3^
Perímetro cefálico*	34,5 (33,5-35,3)	34,5 (33,5-35,5)	34,1 (33,5-35)	0,981^2^
Perímetro torácico**	32,8 (0,3)	32,9 (0,4)	33,1 (0,4)	0,869^3^
Comprimento*	49 (47-50)	49 (47-50)	48,5 (47-49,5)	0,385^2^

* Apresentados em mediana, variação do percentil 25-75;

** média e desvio padrão

1 Teste Exato de Fisher, demais variáveis categóricas analisadas pelo Teste Qui-Quadrado de Pearson;

2 Teste U de Mann-Whitney;

3 Teste T de Student

O sexo do lactente, a ocupação materna, a renda familiar e a anquiloglossia apresentaram associação com o desmame. Entretanto, após ajustamento das variáveis, observou-se que apenas a anquiloglossia presente manteve-se associada com o desmame. A chance de desmame neste grupo foi quase 4,5 vezes maior quando comparados aos lactentes sem a alteração (p < 0,001 OR = 4,49 95% IC = [2,29;8,80]). O fato de ser do sexo feminino como demonstrado na Tabela 2, apresentou valor de P exatamente 0,050, não sendo considerada como fator de proteção por apresentar uma variação do intervalo de confiança muito próxima do 1.

**Tabela 2:** Análise de regressão logística não ajustada e ajustada utilizando o aleitamento materno exclusivo no 6º mês como variável de desfecho e as variáveis expositivas, 2019-2021

	Análise Univariada		Análise Ajustada	
	Valor de P	OR (IC 95%)	Valor de P	OR (IC 95%)
Sexo, feminino	0,042	0,52 (0,28-0,97)	0,050	0,50 (0,25-0,99)
Estado civil, sem comp.	0,192	1,58 (0,79-3,18)		
Ocupação, trabalha	0,043	1,89 (1,02-3,52)		
Renda, entre 3 e 5 SM	0,019	2,58 (1,16-5,69)		
Primiparidade	0,078	0,58 (0,32-1,06)		
Apgar 5º minuto	0,174	0,62 (0,31-1,23)		
Anquiloglossia presente	<0,001	4,50 (2,33-8,67)	<0,001	4,49 (2,29-8,80)

Seleção para modelo ajustado através do método *Stepwise forward*, entrada no modelo com α^1^=0,100 e de saída α^2^=0,050. Variáveis removidas do modelo: estado civil (p = 0,154), ocupação (0,309), Apgar 5 (p = 0,338), renda familiar (0,930) e primiparidade (p = 0,531). Indicadores do ajustamento do modelo p < 0,001, R^2^ 0,0981.

**Legenda:** OR (odds ratio), IC (intervalo de confiança). SM (salários-mínimos)

A Tabela 3 apresenta o risco relativo para o desmame por mês. Observou-se que a partir do segundo mês, os lactentes com anquiloglossia apresentaram risco maior de não estar em AME. De forma geral, a proporção de AME em bebês com a alteração foi sempre menor em todos os meses quando comparados aos lactentes sem a alteração.

**Tabela 3:** Prevalência e estimativas de Risco de Aleitamento Materno Exclusivo dos participantes do estudo, categorizados de acordo com a anquiloglossia, 2019-2021

	N (%)	Anquiloglossia		RR (IC 95%)
		Não	Sim	Valor de P
		n(%)	n(%)	
**Reportou AME**				
1º mês	199 (93)	161 (94,7)	38 (86,4)	2,5 (0,9-6,8)
				0,058
2º mês	157 (89,7)	134 (93,1)	23 (74,2)	3,7 (1,5-8,6)
				0,002
3º mês	146 (88,5)	124 (94,7)	22 (64,7)	6,6 (2,8-15,4)
				<0,001
4º mês	146 (86,9)	125 (91,9)	21 (65,6)	4,25 (2,0-8,9)
				<0,001
5º mês	126 (83,4)	111 (88,8)	15 (57,7)	3,7 (1,9-7,3)
				<0,001
6º mês	95 (49,7)	84 (56,8)	11 (25,6)	1,7 (1,3-2,2)
				<0,001

**Legenda:** RR – risco relative, IC – intervalo de confiança, AME – aleitamento materno exclusivo

A Figura 1 apresenta a diferença das curvas de Kaplan-Meier entre os dois grupos analisados demonstrando que os lactentes com anquiloglossia apresentaram tempo menor de aleitamento (log-rank p < 0,001) com média de 4,5 meses (95%IC = 4,03;4,97) de duração de AME. Sendo que para o grupo sem a alteração a média foi de 5,4 meses (95% IC 5,2-5,6).

**Figura 1:** Curvas de Kaplan-Meier representando o tempo do desmame segundo a presença ou não de anquiloglossia. Os indivíduos com anquiloglossia tiveram um tempo de ocorrência do desmame pior do que os indivíduos que não tinham anquiloglossia (p < 0,001). A média de aleitamento exclusivo (com intervalo de confiança de 95%) para o grupo sem anquiloglossia foi de 5,40 (5,2 - 5,6) meses; e do grupo com anquiloglossia foi de 4,50 (4,03 - 4,97)

## DISCUSSÃO

Os principais achados deste estudo que analisou a influência da anquiloglossia nos aspectos de AME, sugerem que essa alteração está associada com o desmame mais proximal ao nascimento em lactentes a termo saudáveis diagnosticados, quando comparados a lactentes sem a alteração.

A influência da anquiloglossia sobre o tempo de aleitamento reportado neste estudo concorda com alguns relatos semelhantes na literatura médica. Um estudo realizado na Espanha com 1102 recém-nascidos detectou maior prevalência de alterações em meninos e a taxa de aleitamento exclusivo foi de 50,35%^(20)^. Estudo prospectivo realizado em Londres referiu uma taxa de 49% de AME em bebês com anquiloglossia em um seguimento de 3 meses^(21)^. Taxas maiores de adesão ao aleitamento exclusivo, como, por exemplo, de 66,6% aos 3 meses foram relatadas quando os lactentes foram submetidos a procedimentos cirúrgicos de correção previamente^(22)^. Percebe-se em abordagem observacionais que as taxas de AME são semelhantes às encontradas neste estudo.

Estudo referiu que taxas de AME podem ser semelhantes mesmo sem considerar intervenções para correção da alteração^(23)^. É necessário destacar que nas primeiras semanas de vida, a presença da anquiloglossia é concomitante a outras alterações no aleitamento^(18)^. Estudos ainda afirmam que a presença da alteração pode estar relacionada com uma percepção negativa da mãe ao amamentar, o que pode levar a uma dúvida sobre qual fator pode estar gerando as dificuldades nas primeiras semanas de amamentação^(24)^.

Diversos fatores parecem mediar a relação entre alteração do frênulo lingual e desmame precoce, visto que é concomitante a outros problemas precoces como dor e dificuldade de pega, que estão associados com a amamentação nas primeiras semanas de vida e prejudicam o estabelecimento da alimentação natural de recém-nascidos. Ainda, podem ser relacionadas com outras condições prévias como parto cesáreo e ausência de experiência prévia de amamentação^(7,18,25,26)^.

No presente estudo, é importante considerar que associação entre anquiloglossia e desmame se mostrou presente logo a partir do segundo mês de vida da criança, um período crítico em que é frequentemente referido o abandono da amamentação exclusiva devido ao retorno das mães ao trabalho^(27)^. No Brasil, a maioria das mulheres assistidas pela legislação trabalhista geral tem direito à licença maternidade com remuneração até o quarto mês pós-parto, porém deve-se considerar que o trabalho informal é frequente.

De acordo com o Estudo Nacional de Alimentação e Nutrição Infantil^(28)^, a prevalência do AME no Brasil tem demonstrado tendência de crescimento, alcançando 60% das crianças com menos de 4 meses e 45,7% das crianças com menos de 6 meses. Entretanto, a duração mediana do AME foi de 54,1 dias (1,8 meses) de acordo com a última pesquisa de prevalência de aleitamento materno^(29)^. Os indicadores de aleitamento reportados neste estudo podem demonstrar a importância do suporte à mulher, dando enfoque a identificação das mães com risco da descontinuação precoce^(30)^. As ações praticadas nos bancos de leite parecem ter efeito positivo na promoção do aleitamento e no suporte aos bebês que apresentam dificuldade para mamar no seio^(31)^.

Esta pesquisa foi conduzida num centro especializado em aleitamento materno, o que pode levar a um viés de seleção ou nas taxas relatadas, visto que as mães eram continuamente orientadas a prosseguir na amamentação exclusiva e eram auxiliadas quando reportavam problemas. Entretanto, esta situação pode ser encarada como ponto forte visto que, ainda para o grupo com a alteração, o tempo total de aleitamento foi maior que a média nacional, demonstrando que um adequado acompanhamento e orientação podem ser determinantes para a diminuição da chance de desmame, destacando-se, portanto, as medidas clínicas e não cirúrgicas como efeito protetor do aleitamento.

A frenotomia tem sido apontada como tratamento padrão para diagnóstico positivo da alteração com casos sintomáticos, reduzindo principalmente a dor perceptível pelas mães^(32)^. Entretanto, é necessário que haja uma avaliação clínica precisa, consenso entre profissionais de saúde que lidam com a amamentação, baseada nos melhores protocolos científicos^(19,33)^, a fim de evitar procedimentos desnecessários e evitar iatrogenias^(5)^.

Outra dificuldade deste estudo está relacionada ao seguimento dos pacientes, apontando-se como uma das limitações, visto que a participação voluntária e o nível socioeconômico das mães podem ter impedido um maior comparecimento às consultas de seguimento. Ademais, as díades que estavam com sucesso no aleitamento podem não ter sentido necessidade de comparecimento ao seguimento, ainda que tivessem consulta médica agendada. Novos estudos devem ser encorajados para estabelecer o impacto tardio da alteração anatômica na língua e mensuração de indicadores objetivos para verificar alterações de sucção e deglutição nesses bebês. Destaca-se que este estudo pode servir de base para outras observações que levem em conta fatores que possam atuar junto com a anquiloglossia e que podem interferir no aleitamento materno. Salientamos ainda para a necessidade de o fonoaudiólogo ser parte indispensável da equipe multiprofissional, pois é o profissional habilitado para avaliar e tratar as disfunções orais presentes no aleitamento materno.

## CONCLUSÃO

Neste estudo, a anquiloglossia neonatal demonstrou ter uma associação sobre a alimentação em lactentes a termo com anquiloglossia do tipo assintomática (sem necessidade de intervenção) em comparação com lactentes com freio lingual normal, reduzindo o tempo de exclusividade do aleitamento, aumentando o risco de desmame.
